# Effects of a 2-Week Remote Learning Program on Empathy and Clinical and Communication Skills in Premedical Students: Mixed Methods Evaluation Study

**DOI:** 10.2196/33090

**Published:** 2021-10-27

**Authors:** Ujwal Srivastava, Amy Price, Larry F Chu

**Affiliations:** 1 Anesthesia, Informatics and Media Lab Stanford School of Medicine Palo Alto, CA United States

**Keywords:** empathy, clinical skills, communication skills, high school, undergraduate, summer program, premedical program, remote learning

## Abstract

**Background:**

Expressing empathy builds trust with patients, increases patient satisfaction, and is associated with better health outcomes. Research shows that expressing empathy to patients improves patient adherence to medications and decreases patient anxiety and the number of malpractice lawsuits. However, there is a dearth of research on teaching empathy to premedical students. The Clinical Science, Technology, and Medicine Summer Internship of Stanford Medicine (also called the Stanford Anesthesia Summer Institute) is a 2-week collaborative medical internship for high school and undergraduate students to inspire learners to be compassionate health care providers. The summer 2020 program was adapted to accomplish these objectives in a fully remote environment because of the COVID-19 global pandemic.

**Objective:**

This study aims to measure the change in empathy and competencies of participants in clinical and communication skills before and after program participation.

**Methods:**

A total of 41 participants completed only the core track of this program, and 39 participants completed the core + research track of this program. Participants in both tracks received instructions in selected clinical skills and interacted directly with patients to improve their interviewing skills. Research track participants received additional instructions in research methodology. All participants completed web-based pre- and postsurveys containing Knowledge and Skills Assessment (KSA) questions. Participant empathy was assessed using the validated Consultation and Relational Empathy measure. A subset of participants completed optional focus groups to discuss empathy. The pre- and post-KSA and Consultation and Relational Empathy measure scores were compared using paired 2-tailed *t* tests and a linear regression model. Open-ended focus group answers were then analyzed thematically.

**Results:**

Participants in both tracks demonstrated significant improvement in empathy after the 2-week remote learning course (*P*=.007 in core track; *P*<.001 in research track). These results remained significant when controlling for gender and age. A lower pretest score was associated with a greater change in empathy. Participants in both tracks demonstrated significant improvement in KSA questions related to surgical skills (*P*<.001 in core track; *P*<.001 in research track), epinephrine pen use (*P*<.001 in core track; *P*<.001 in research track), x-ray image interpretation (*P*<.001 in core track; *P*<.001 in research track), and synthesizing information to solve problems (*P*<.001 in core track; *P*=.05 in research track). The core track participants also showed significant improvements in health communication skills (*P*=.001). Qualitative analysis yielded 3 themes: empathy as action, empathy as a mindset, and empathy in designing health care systems.

**Conclusions:**

Summer internships that introduce high school and undergraduate students to the field of health care through hands-on interaction and patient involvement may be an effective way to develop measurable empathy skills when combined with clinical skills training and mentorship. Notably, increases in empathy were observed in a program administered via a remote learning environment.

## Introduction

### Benefits of Empathy for Patients and Physicians

Increased physician empathy leads to better patient outcomes [1,2]. In studies of patients with diabetes and the common cold, patients of physicians with high levels of empathy had better clinical outcomes and quicker recovery [3,4]. Higher levels of patient-perceived empathy in physicians have been correlated with improved patient adherence to medication regimens [1,5], which remains a pressing issue in reducing complications and promoting health outcomes [6]. It is suggested that these benefits stem from strong communication and mutual trust and understanding in the patient-physician relationship, all of which can be promoted by physicians expressing empathy [1,7]. Crucially, having greater physician empathy has been linked with higher patient satisfaction and trust [5,8], which can lead to patients disclosing more detailed information, more accurate diagnoses, and shared decision-making [9]. Empathy in physicians also decreases patient anxiety [10] and increases patient enablement [11]. Furthermore, quality of care improves because more empathic physicians have fewer malpractice claims [12] and are less likely to commit medical errors [13].

Empathy can also impact caregivers. Those found to have higher empathy for patients also have higher job satisfaction and well-being [14]. High empathy might be a protective factor against the growing and worrying trend of physician burnout. More than half of the physicians now report symptoms of burnout, including emotional exhaustion, depersonalization, and lack of accomplishment [15]. The consequences of burnout include higher rates of medical errors and lower patient satisfaction. A systematic review of the relationship between empathy and burnout found a negative correlation in 8 out of 10 studies [16].

### Empathy in Medical Education

Despite these studies on the positive impact of empathy in medicine, there is a dearth of research on how to cultivate empathy in medical education. Our scope of research is motivated by this overarching hypothesis: increases in empathy during early medical education could be crucial in preventing burnout in medical school later [17]. If increases in empathy persist past medical training, they could lead to improved clinical outcomes. Our review of the literature found some studies on empathy in the medical student population; however, the research findings are mixed. Although many studies found that female medical students have significantly higher empathy scores than male students [18-21], some studies did not find such associations [22,23]. Studies examining changes in empathy across the course of medical school have concluded that empathy scores tend to decline across the years of medical school, with younger medical students in preclinical years exhibiting significantly higher empathy scores [17,19-22]; however, there is no systemic evidence for this decline [9]. Recent research also indicates that students who enter medical school at an older age are significantly more likely to demonstrate higher empathy than students entering at a younger age [21]. These findings regarding factors that influence empathy need to be further corroborated.

In the premedical student population, several summer premedical programs for high school and undergraduate students exist, but no studies have focused on empathy in this population. Common outcomes for evaluating these programs include tracking how many students pursue degrees and careers in medicine [24,25], assessing students’ attitudes toward science and students’ mastery in various program goals such as scientific literacy and laboratory skills [26-28]. Tools for program evaluation include self-assessment Likert surveys and open-ended questionnaires. Prior programs do not report cultivating a mindset of compassion or the use of empathy as a primary outcome measure of program efficacy. Therefore, we decided to conduct this mixed-methods study of a 2-week remote learning premedical program and its effects on empathy and clinical and communication skills. This study evaluates the stated program to determine whether there were changes in empathy and competencies in selected clinical skills among program participants.

## Methods

### Curriculum Development

The Clinical Science, Technology, and Medicine Summer Internship (also called the Stanford Anesthesia Summer Institute [SASI]) is a 2-week program for high school and undergraduate students run by the Anesthesia, Informatics, and Media laboratory at the Stanford School of Medicine. The program began in June 2017 and has run every year since, with 435 program participant graduates. Program instruction is typically held in person on the Stanford School of Medicine campus, but the summer 2020 curriculum was adapted to be fully remote because of the COVID-19 pandemic.

The SASI Core curriculum was co-designed with input from patients, high school science teachers, anesthesiology professors, and researchers. Key features of the curriculum include opportunities for SASI participants to interact directly with patients; improve clinical skills through hands-on training; and receive mentorship from clinicians, patients, and near peers. Students take part in lectures and workshops hosted by patients, medical students, and faculty on topics such as empathic listening, emergency medicine, and careers in medicine. They also learned the principles of Everyone Included [29] and worked on a capstone project to coproduce a health care solution with their e-patient [30]—a term used to describe engaged patients who play an active role in their health care decision-making process. Core track participants only attend the morning session, which covers the core curriculum. Research track participants attend the morning session and additional afternoon sessions that focus on research methodology.

### Study Design

**Figure 1:**
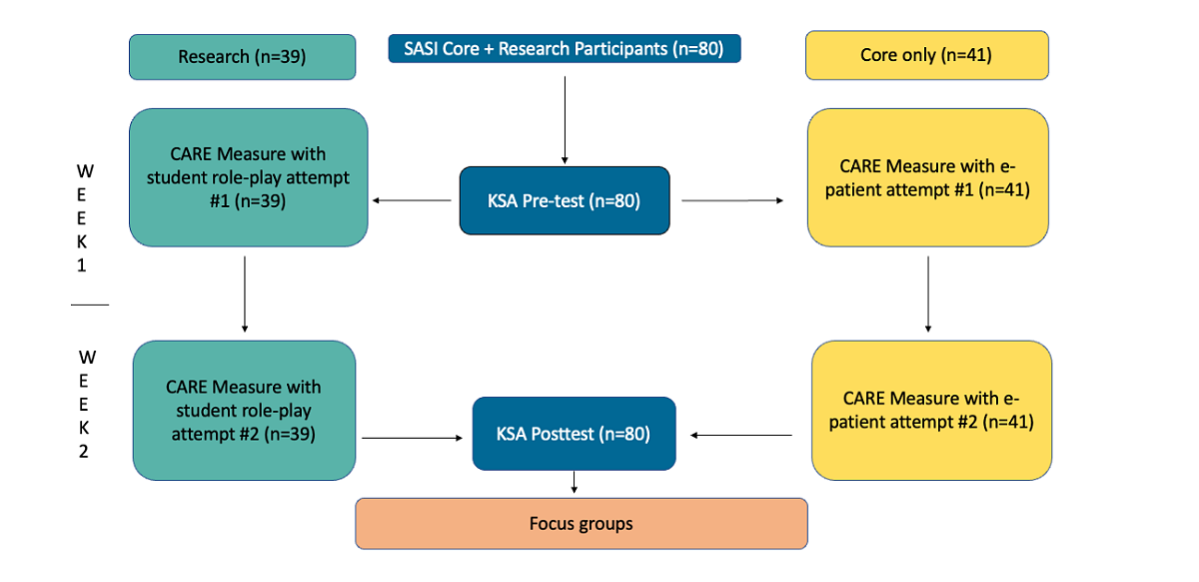
Study design. Data are collected using a pre-post format for the 2 tracks separately. CARE: Consultation and Relational Empathy; KSA: Knowledge and Skills Assessment; SASI: Stanford Anesthesia Summer Institute.

Although SASI has run before, we developed a novel pilot study to assess empathy in participants (Figure 1). Patients were involved in the coproduction of the study design methodology, with special consideration to patient inclusion, participatory methods, and the inclusion of Everyone Included principles [31].

“Everyone Included™ creates a culture of health in which everyone is trusted and respected for the expertise they bring, where openness and experimentation is the norm, people have personal ownership of health, individual stories have global impact, and the patient voice and choice is a part of all stakeholder decisions” [29].

All SASI participants completed pre- and postsurveys that included Knowledge and Skills Assessment (KSA) questions and questions about attitudes toward empathy. Participant empathy was assessed using the validated Consultation and Relational Empathy (CARE) measure. Core track participants were evaluated by e-patients, and research track participants were evaluated by peers during role-play. Participants were invited to complete an optional focus group at the end of the program to discuss empathy. The purpose of this study design was to evaluate the program by comparing each participant with themselves before and after program completion; thus, they acted as their own control.

Our study protocol was evaluated by the Stanford Institutional Review Board, and it was determined that it does not meet the definition of research, as defined in 45 CFR 46.102(d).

Classroom interactions were conducted in accordance with the Stanford policies outlined in the Guidelines for Online Minors Programs developed by the Office for Protection of Minors. This includes appropriate staff training.

### Participants

High school and undergraduate students were selected to participate in SASI through an application process. Accepted program participants were invited to participate voluntarily in our evaluation study. A total of 41 participants were in core track only, and 39 participants were in research track.

### Materials

#### CARE Measure

The CARE measure is regarded as one of the best-validated patient rating scales of practitioner empathy [32]. The survey consists of 10 Likert scale items that aim to evaluate the human aspects of medical care, with a focus on the process rather than the outcome [33]. It has specifically been cited as a potential tool for assessing empathy in undergraduate medical education [33]. All reviewers filled out an electronic version of the survey (Multimedia Appendix 1 [33,34]). The CARE measure was scored by the authors using an official scoring system [33].

#### Standardized Role-play

Participants took turns role-playing as physicians and patients during a mock medical history–taking session. Those playing the patient were given a specific patient profile card, and those playing the physician were given a medical history–taking form. All scenarios and materials were adapted from the Association of American Medical Colleges premed lesson plans [35]. After the mock consultation, empathy of the participant who played the physician was assessed using the CARE measure.

#### SASI Survey

The SASI survey was developed by the authors to be electronically completed before and after program participation. Specifically, the survey included demographic information (gender, grade, race, and program track), questions about attitudes toward empathy, the Likert-scale KSA questions (1: strongly disagree; 2: disagree; 3: neither agree nor disagree; 4: agree; and 5: strongly agree), and program reflection questions (in the postsurvey only; Multimedia Appendices 2 and 3).

#### Qualitative Feedback

SASI participants had the option of completing focus groups to explore empathy. Open-ended questions presented various scenarios regarding empathy (Multimedia Appendix 4).

#### Zoom Platform

The SASI curriculum instruction was imparted synchronously over the Zoom videoconferencing platform (Zoom Video Communications). Efforts to maintain an engaging sense of community included encouraging participants to keep cameras on, frequent use of breakout rooms, and icebreakers and breaks to prevent Zoom fatigue.

### Data Collection and Analysis

#### Overview

Data were collected according to the following timeline. Day 1 refers to the first day of SASI. All program participants and e-patients were given deidentified program IDs so that they could fill out all surveys anonymously. All quantitative data analyses were performed using RStudio (version 4.0.3).

#### Empathy Scores (Quantitative)

##### Day 1

Core track participants met with their assigned e-patient for the first time to complete a pseudohistory–taking session. e-Patients filled out core track CARE Measure Attempt #1 based on their interactions.

Research track participants met in groups of 6 to 8 to complete the role-play activity. In pairs, the participants took turns playing both the physician and patient, according to the character card they were given. For each participant who played the physician, the other participants in the group filled out research track CARE Measure Attempt #1 to assess the physician’s empathy based on the mock consultation. Each physician’s CARE measure scores were averaged to increase validity and consistency and produce a single empathy prescore.

##### Day 6

Core track participants met with the same assigned e-patient again and worked on coproducing their health solutions. e-Patients filled out core track CARE Measure Attempt #2 based on their interactions.

##### Day 9

Research track participants met in the same groups to complete the role-play activity. In pairs, participants repeated the role-play activity, but they were assigned different case scenarios. For each participant who played the physician, the other participants in the group filled out research track CARE Measure Attempt #2 to assess the physician’s empathy based on the mock consultation. Each physician’s CARE measure scores were averaged to increase validity and consistency and produce a single empathy prescore.

For hypothesis testing of CARE measure scores, normality of change in scores was assessed using a quintile plot, and boxplots were generated to find outliers. Initial testing was performed using paired 2-tailed *t* tests by track. Follow-up analysis was performed using multivariate linear regression to control for the effects of gender, grade, and pretest empathy score on changes in empathy. We met the guidelines for minimum sample size [36].

#### KSA Scores (Quantitative)

##### Day 1

SASI participants filled out the SASI presurvey to self-assess their knowledge and skills.

##### Day 9

SASI participants filled out the SASI postsurvey to self-assess their knowledge and skills.

For hypothesis testing of KSA questions, normality of change in scores was assessed using a quintile plot and boxplots were generated to find any outliers. Paired *t* tests were performed for each question by track.

#### Focus Groups (Qualitative)

Subset of SASI participants completed focus groups on Day 10.

Qualitative methods were guided by the Grounded Theory of Strauss and Corbin [37]. Focus group responses were transcribed using Descript software. Transcripts were read over for accuracy and data familiarization. We used open and axial coding to conduct qualitative analysis of the focus group responses. A researcher (US) read the responses and identified major themes and subthemes based on the frequency of repetition of keywords. Quotes that supported the generated themes were selected to highlight the anecdotes. Final themes were reviewed by an independent researcher (LFC) to ensure accuracy and consistency.

#### Overall Program Feedback (Qualitative)

SASI participants filled out the SASI postsurvey to provide overall program feedback on Day 9.

Responses were read and analyzed by the authors, and quotes were selected to highlight the anecdotes.

## Results

### Descriptive Statistics

A total of 90 participants completed the SASI program, of which 80 participants completed the SASI pre- and postsurveys that matched (core track, n=41 and research track, n=39). Of these 80 participants, 55 participants had complete CARE measure data for both attempts that matched (core track, n=20 and research track, n=35). Table 1 presents demographic data of all the participants who participated in the study. There were no significant differences in age, gender, and race in the subgroup that completed the CARE measure data surveys (chi-square test: *P*=.94 in core track and *P*=.99 in research track).

**Table 1:** Participant demographic data (n=80).

Characteristic		Participants in core track (n=41), n (%)	Participants in research track (n=39), n (%)
**Gender**			
	Male	15 (37)	16 (41)
	Female	26 (63)	21 (54)
	Prefer not to say	0 (0)	2 (5)
**Grade**			
	High school student	36 (88)	34 (87)
	College student	5 (12)	5 (13)
**Race**			
	African American	2 (5)	1 (3)
	Asian	27 (66)	29 (74)
	White	3 (7)	5 (13)
	More than one race	5 (12)	0 (0)
	Other	3 (7)	2 (5)
	Prefer not to say	1 (2)	2 (5)

### Empathy Scores (Quantitative)

Participants in both tracks demonstrated significant improvement in empathy after the 2-week remote learning course, as assessed by paired *t* tests. In the core track (n=20), the mean CARE score increased from 31.31 (SD 10.81) before SASI to 40.75 (SD 12.57) after SASI (*P*=.007). In the research track (n=35), the mean CARE score increased from 40.42 (SD 6.24) before SASI to 43.50 (SD 3.76) after SASI (*P*<.001). The CARE score ranges from 10-50. Each Likert-scale question is scored 1 (poor) to 5 (excellent), and there are a total of 10 questions.

### Effect of Gender, Grade, and Pretest Score on Change in Empathy

To further study changes in empathy scores, we used multivariate linear regression analysis to model differences in pre- and posttest empathy scores as the dependent variable, adjusting for gender, grade, and empathy pretest score.

For the research track, improvement in empathy scores remained statistically significant after controlling for gender and grade. Participants with lower CARE measure pretest scores were significantly more likely to improve their empathy scores (Table 2). For core track, improvement in empathy scores remained statistically significant after controlling for gender, grade, and empathy pretest scores (Table 3).

**Table 2:** Multivariate linear regression model for factors influencing change in the Consultation and Relational Empathy (CARE) measure pre- and posttest scores in Stanford Anesthesia Summer Institute research track participants.^a^

Factors	β coefficient (SE)	*P* value
Intercept	24.337 (2.741)	<.001
CARE measure pretest score	−0.534 (.068)	<.001
Gender (female)	0.891 (.842)	.30
Gender (prefer not to say)	2.524 (1.833)	.18
College student	−1.822 (1.191)	.14

^a^Adjusted *R*^2^=0.668.

**Table 3:** Multivariate linear regression model for factors influencing change in the Consultation and Relational Empathy (CARE) measure pre- and posttest scores in Stanford Anesthesia Summer Institute core track participants.^a^

Factors	β coefficient (SE)	*P* value
Intercept	27.167 (8.803)	.007
CARE measure pretest score	−0.587 (0.283)	.05
Gender (female)	−1.612 (6.478)	.81
College student	11.963 (7.656)	.14

^a^Adjusted *R*^2^=0.236.

### KSA Scores (Quantitative)

Participants in both tracks demonstrated significant improvement in the total KSA score after program completion. In core track, the mean score increased from 27.93 (SD 4.06) before SASI to 34.51 (SD 3.04) after SASI—a 24% improvement (*P*<.001). Significant increases were specifically observed in KSA questions 1-5. In research track, the mean score increased from 29.28 (SD 2.96) before SASI to 35.33 (SD 3.26) after SASI—a 21% improvement (*P*<.001). Significant increases were specifically observed in KSA questions 1-4. Table 4 summarizes the KSA score changes before and after program completion by track for each KSA question.

**Table 4:** Paired *t* test for Knowledge and Skills Assessment (KSA) scores by question before and after the completion of the Stanford Anesthesia Summer Institute internship (n=80).

Health outcome	Participants in core track (n=41)					Participants in research track (n=39)			
	Pretest, mean (SD)	Posttest, mean (SD)	Change (%)	*P* value	Pretest, mean (SD)		Posttest, mean (SD)	Change (%)	*P* value
KSA question 1^a^	2.05 (1.28)	3.95 (0.77)	92.7	<.001	2.62 (1.35)		4.21 (0.52)	60.7	<.001
KSA question 2^b^	2.34 (1.54)	4.51(0.55)	92.7	<.001	1.95 (1.05)		4.51 (0.51)	131.3	<.001
KSA question 3^c^	2.07 (1.14)	3.37 (1.04)	62.8	<.001	2.05 (0.92)		3.56 (0.88)	73.7	<.001
KSA question 4^d^	3.83 (0.59)	4.49 (0.51)	17.2	<.001	4.33 (0.62)		4.51 (0.60)	4.2	.05
KSA question 5^e^	3.93 (0.87)	4.41 (0.55)	12.2	.001	4.33 (0.70)		4.56 (0.60)	5.3	.08
KSA question 6^f^	4.32 (0.69)	4.46 (0.60)	3.2	.18	4.54 (0.60)		4.62 (0.49)	1.8	.50
KSA question 7^g^	4.63 (0.58)	4.61(0.44)	−0.4	.80	4.64 (0.49)		4.67 (0.48)	0.6	.71
KSA question 8^h^	4.76 (0.43)	4.71 (0.46)	−1.0	.57	4.82 (0.39)		4.69 (0.47)	−2.7	.10

^a^Apply the basic surgical suture knot (1 [strongly disagree] to 5 [strongly agree]).

^b^Apply an epinephrine pen to a person with anaphylaxis (1 [strongly disagree] to 5 [strongly agree]).

^c^Interpret the basic chest x-ray for signs of pneumothorax, hemothorax, or major trauma (1 [strongly disagree] to 5 [strongly agree]).

^d^Analyze relevant information and arrive at a solution to a new challenge (1 [strongly disagree] to 5 [strongly agree]).

^e^Demonstrate effective communication skills to promote health (1 [strongly disagree] to 5 [strongly agree]).

^f^Connect and expand on ideas when collaborating with peers (1 [strongly disagree] to 5 [strongly agree]).

^g^Listen to a friend sharing their problem (1 [strongly disagree] to 5 [strongly agree]).

^h^Role of technology in solving health care challenges (1 [strongly disagree] to 5 [strongly agree]).

### Focus Groups (Qualitative)

#### Overview

A total of 32 participants consented to be recorded in the focus groups. Three main themes were constructed from transcript data. These themes are elaborated here using brief explanations and verbatim quotations. The major themes established after reading all transcripts are presented as follows:

1. Empathy as action.
2. Empathy as a mindset.
3. Empathy in designing health care systems.

#### Theme 1: Empathy as Action

This theme includes responses that characterize empathy as a set of specific actionable items that a physician can implement, such as listening to their patient; appropriate use of voice, tone, and body language when conversing; and fine-tuning treatment plans according to patient needs to yield better adherence and outcomes:

Empathy is being a good listener, not portraying judgement, and being open to change in terms of what you might not know and expanding your knowledge set.

Letting [patients] tell their side of the story and believing that there’s something there and not dismissing their concerns...the body language of the doctor and things like not commanding [patients] shows if they’re engaged with the patient or not.

#### Theme 2: Empathy as a Mindset

This theme includes responses that characterize empathy as a mindset that physicians can adopt to be more empathetic toward patients. These responses focused on ways to undo biases physicians may have, with potential solutions such as metaphorically putting oneself in the patient’s shoes and treating them as a whole person:

In the doctor-patient context, empathy is what makes the patient feel they are considered as human beings instead of being analyzed by their diseases.

A lot of patients were saying that they like to be looked at as more than their disease or their diagnosis, and to be looked at as a whole person by their doctor.

#### Theme 3: Empathy in Designing Health Care Systems

Although previous responses centered on the individual responsibilities of the physician, responses in this theme were related to empathy in the context of the health care system as a whole. The predominant suggestion was to encourage empathy via shared decision-making, including giving the patient an equal voice and not being ignorant of or condescending toward the patient:

Without empathy, I feel like we might easily fall into a philosophy of considering efficiency when designing healthcare, and not actually consider the patients as humans.

The clinician should be in the driver’s seat and the patient should be in the passenger seat, not in the back. Empathy is about making sure that everyone has a say. Traditional medicine focuses on this idea that the patient just listens to a doctor and does they say, but patients have their own experiences.

### Program Feedback (Qualitative)

In the postsurvey, participants were asked to rate their overall program experience (1: poor; 2: fair; 3: good; 4: very good; and 5: excellent). SASI was well-received and earned an average score of 4.04 (SD 0.87).

Participants were also asked to respond to open-ended, written questions about the overall program. Common themes were identified and are presented later with verbatim quotations.

Three major themes were coded in response to the question *What was the most important thing you learned as a SASI student?* The 3 themes are as follows: 41% (33/80) of the participants referenced empathy in their answers, 31% (25/80) referenced patients and health care systems, and 24% (19/80) referenced specific clinical skills such as suturing and basic life support:

The most important lesson I learned is to have empathy for patients. Before SASI, I knew that physicians should treat patients with empathy, but I did not know how to apply the practice of empathy or about the perspective of patients. However, through this program, I had the opportunity to hear from patients about their stories, which gave me a better understanding of how to treat people with empathy.

When asked to identify their favorite lecture or activity, 64% (51/80) of participants selected the “10 Ways to Die” lecture—a crash course on the leading causes of mortality in the United States—or the suturing activity—one of the few hands-on activities made possible during the remote learning program by mailing a suturing kit to all participants. Participants described these activities as “engaging,” “practical,” and “fun.”

Most participants shared that the SASI had increased their motivation and interest in pursuing a career in health care. In terms of criticism, the most common feedback was difficulties associated with remote learning.

## Discussion

### Principal Findings

This study evaluated a 2-week remote learning premedical program and found that it was successful in achieving its course objectives. Quantitative analysis of pre- and posttest scores indicated that participant empathy and clinical and communication skills increased after program completion. Qualitative analysis of open-ended responses suggested that SASI developed a participant’s understanding of empathy at the individual and systemic level. Overall, the program was well-received, although remote learning posed some challenges.

The CARE measure score data support that clinically relevant empathy can be taught in a remote learning environment. SASI participants had measurable improvements after program completion: the mean CARE measure score increased by 9 points in core track and by 3 points in research track. The CARE measure is composed of 10 questions (scored 1-5), each assessing a unique subskill that builds toward physician empathy. Thus, a 3-point improvement can roughly be equated to a 1 Likert-level improvement in 3 of the 10 empathy skills assessed in the CARE measure. Similarly, a 9-point increase represents a holistic improvement in multiple facets of the CARE measure. In terms of absolute scores, a few studies have published benchmark means that provide a reference comparison. While developing and testing the CARE measure, the designers concluded an average score of 40.8 [34]. By this standard, research track went from below average (40.42) to above average (43.50) during the program and core track progressed from severely below average (31.31) to average (40.75). One possible explanation for the low core track CARE measure prescore could be the initial hesitation to talk to an unknown adult patient. A meta-analysis of 64 studies involving the CARE measure found a mean score of 40.42 [32]. By this metric, both tracks finished with above-average CARE measure scores. The CARE measure has been credited for its robustness and relevancy by both patients and physicians and has demonstrated strong validity across multiple patient populations and settings [34].

The CARE measure provides an external rating for patient-assessed empathy, and the thematic analysis of the focus groups corroborates these findings from the participant’s perspective. Participant responses demonstrate that they have inculcated a mindset of empathy (theme 2) and have the requisite tools to execute their empathy meaningfully (theme 1). They also have a broader view of the importance of empathy (theme 3), which will hopefully sustain empathy.

The KSA data reveal measurable gains in self-assessed clinical and communication skills. Participants in both tracks demonstrated significant improvement in KSA questions related to surgical skills, epinephrine pen use, x-ray image interpretation, and synthesizing information to solve problems (questions 1-4). The core track participants also showed a significant improvement in health communication skills (question 5). The high baseline health communication score could be a reason why the improvement was not significant in the research track participants. Questions 6 and 7 reference domains of broad, transferable skills that, although related to the field of medicine, are not necessarily specific to that domain. Therefore, premedical students were likely to have had prior knowledge or skill development in these domains. This could explain the high baseline scores observed for questions 6 and 7 in our student cohort. High baseline scores could be a reason why there was no significant improvement in either track in questions relating to collaborating and listening to peers. Despite the SASI curriculum advocating for technology to promote health care, it is interesting to note that support for technology had a slight decrease in both tracks. Given that participants had an increase in empathy, this supports previous research that found a negative correlation between empathy and medical students who preferred technology-oriented specialties [18].

There are a few key SASI program features that facilitate teaching empathy. Most notably, SASI creates opportunities for early patient interactions. SASI students participate in e-patient–led lectures and workshops such as *Empathic Communication and Leadership in Healthcare*, co-design health solutions with e-patients, and practice interviewing skills with them via standardized consultations. In previous research, early patient contact has repeatedly been cited as a motivator for empathy in medical students [38-41]. SASI is taking a novel approach to extend this a step further by introducing patient contact as early as in high school and undergraduate years of college. The role-play activity is crucial in improving health communication and allowing students to perceive both patient and physician perspectives, and this tool has been found to boost empathy in medical students [42]. In addition, the SASI curriculum emphasizes empathy as a priority; allows for self-reflection through guided activities such as focus groups; facilitates clinical and communication skills training; and provides positive role models and mentorship from physicians, teaching staff, and near peers. All of these factors have been found to promote empathy [38].

It is worth noting that the most popular activities, as rated by SASI participants, were hands-on clinical skills training and a Socratic-style discussion. This can be attributed to the remote environment, in which opportunities for engagement lead to higher ratings. The charisma of the speaker or the chance to get a feeling of a clinical experience can excite students, but it is equally important to include the empathy-enhancing activities described earlier in the curriculum.

This is one of the several issues with the remote learning environment that participants mentioned in the postsurvey. They also noted a lack of engagement with peers and speakers (both socially and professionally), difficulties staying energized for extended hours on Zoom, occasional troubles with time zones and logistics, and fewer hands-on opportunities. Despite these criticisms, significant improvements in empathy were observed in the remote setting.

### Comparison With Prior Work

In our study, the increase in empathy was not affected by gender, as has been the case in previous studies [18-21]. Initial age did not affect the ability to improve empathy, as previously proposed [21]. The regression analysis that measured the effect of gender and age had differing *R*^2^ values for the 2 tracks. A low *R*^2^ value in core track suggests that the independent variables might be less predictive of change in the outcome, and there could be other independent variables that could be studied further. However, this does not weaken support for the findings that female and male participants benefited equally from SASI, although it is possible that there were baseline empathy differences that persisted even though there was a change in empathy because SASI was the same. Owing to the short nature of the program, we were not able to measure empathy across multiple years of education.

One study found an increase in cultural empathy in college and master’s students after a virtual simulation [43], and another study found videoconference communication training to help build patient trust [44]. Our study evaluated a niche program that uses remote learning to build clinically relevant empathy in high school participants. At the high school level, premedical programs report increased scientific skills and desire to pursue careers in medicine [24-28], both of which were found in SASI participants. Some programs track success through the number of participants who complete degrees in medicine, but we do not have access to such data for SASI currently.

### Limitations

One of the main limitations of this study is that the generalizability of the findings may be limited because of (1) the small sample size of participants, (2) a self-selecting group who voluntarily attended SASI, and (3) lack of a true control group. The main purpose of this study is to report on early work at SASI and assess the program for quality improvement, which is why only SASI participants were included. Another potential limitation is that empathy increases with the familiarity of meeting the same patient over time. This could be addressed by future work that provides a control group who does not participate in the SASI curriculum but meets the same patient multiple times. In addition, the KSA scores were self-assessed, which could introduce potential bias. In addition, there was only one coder for the interview data. To reduce the effect of this potential bias, the themes and quotes were discussed among the authors.

### Future Work

The results of this study are promising and indicate that the effects of SASI should be studied further. Future work should focus on the next in-person iteration of the program to see how empathy and clinical and communication skills change during an in-person SASI session. Future work should also target longitudinal analysis to see how SASI participants fare over time. Initially, we could measure how many participants went into medical careers, track these participants, and correlate findings with clinical outcomes. The CARE measures could also be examined. We hypothesize that the increase in empathy demonstrated in the CARE measure scores of SASI participants could translate to meaningful impacts in actual clinical outcomes, as premedical students progress to clinical practice. It remains to be seen whether the empathy gains of SASI translate to meaningful clinical metrics such as better patient satisfaction, less burnout, and fewer medical mistakes. This analysis could also assess whether the SASI empathy effect persists through medical school or whether the benefits are only of short term. If this is the case, follow-up SASI workshops to boost empathy during medical school could be explored.

### Conclusions

The findings of this study indicate that summer premedical internships for high school and undergraduate students are well-received and can inspire participants to pursue careers in health care. Program participation can lead to increased patient-assessed empathy and significant improvement in self-assessed clinical skills, even in a web-based, remote learning environment. Key program features that enable these benefits include early patient contact, role-play activities, and hands-on clinical and communication skills training and mentorship.

## Appendix

Multimedia Appendix 1 Consultation and Relational Empathy measure.

Multimedia Appendix 2 The Stanford Anesthesia Summer Institute presurvey (including Knowledge and Skills Assessment questions).

Multimedia Appendix 3 The Stanford Anesthesia Summer Institute postsurvey (including Knowledge and Skills Assessment questions).

Multimedia Appendix 4 Focus group questions.

## Abbreviations

CARE: Consultation and Relational Empathy
KSA: Knowledge and Skills Assessment
SASI: Stanford Anesthesia Summer Institute
