# Supplementary material for: Effects of a 2-Week Remote Learning Program on Empathy and Clinical and Communication Skills in Premedical Students: Mixed Methods Evaluation Study
Source: JMIR Med Educ. 2021 Oct 27;7(4):e33090. doi: 10.2196/33090 (PMC8581748; doi:10.2196/33090)
Supplement: Multimedia Appendix 1 [file mededu_v7i4e33090_app1.pdf]

The CARE Measure

© Stewart W Mercer 2004

1.Please rate the following statements about today's consultation. Please tick one box for each statement and <u>answer every statement</u>.

How was the doctor at …	**Poor**	**Fair**	**Good**	**Very Good**	**Excellent**	**Does Not Apply**
**1. Making you feel at ease……** *(being friendly and warm towards you, treating you with respect; not cold or abrupt)*	□	□	□	□	□	□
**2. Letting you tell your " story"……** *(giving you time to fully describe your illness in your own words; not interrupting or diverting you)*	□	□	□	□	□	□
**3. Really listening ……** *(paying close attention to what you were sayings; not looking at the notes or computer as you were talking)*	□	□	□	□	□	□
**4. Being interested in you as a whole person …** *(asking/knowing relevant details about your life, your situation; not treating you as "just a number")*	□	□	□	□	□	□
**5. Fully understanding your concerns……** *(communicating that he/she had accurately understood your concerns; not overlooking or dismissing anything)*	□	□	□	□	□	□
**6. Showing care and compassion….** *(seeming genuinely concerned, connecting with you on a human level; not being indifferent or "detached")*	□	□	□	□	□	□
**7 . Being Positive……** *(having a positive approach and a positive attitude; being honest but not negative about your problems)*	□	□	□	□	□	□
**8. Explaining things clearly……..** *(fully answering your questions, explaining clearly, giving you adequate information; not being vague*	□	□	□	□	□	□
**9. Helping you to take control**…… *(exploring with you what you can do to improve your health yourself; encouraging rather than "lecturing" you)*	□	□	□	□	□	□
**10. Making a plan of action with you …** *(discussing the options, involving you in decisions as much as you want to be involved; not ignoring your views)*	□	□	□	□	□	□

Information

The Consultation and Relational Empathy (CARE) Measure is a consultation process measure has that been developed by Dr Stewart Mercer and colleagues in the Departments of General Practice at Glasgow University and Edinburgh University. It is based on a broad definition of empathy in context of a therapeutic relationship within the consultation. The wording reflects a desire to produce a holistic, patient-centred measure that is meaningful to patients irrespective of their social class, and has been developed and applied in over 3,000 general practice consultations in areas of high and low deprivation in the west of Scotland.

The scoring system for each item is 'poor'=1, 'fair' = 2, 'good' = 3, 'very good' = 4, and 'excellent'= 5. All ten items are then added, giving a maximum possible score of 50, and a minimum of 10. Up to two 'Not Applicable' responses or missing values are allowable, and are replaced with the average score for the remaining items. Questionnaires with more than two missing values or 'Not Applicable' responses are removed from the analysis.

The theoretical background and validation of the CARE measure can be found in:

Mercer SW, McConnachie A, Maxwell M, Heaney DH, and Watt GCM. Relevance and performance of the Consultation and Relational Empathy (CARE) Measure in general practice. Family Practice 2005, 22 (3), 328-334

Mercer SW, Watt, GCM, Maxwell M, and Heaney DH. The development and preliminary validation of the Consultation and Relational Empathy (CARE) Measure: an empathy-based consultation process measure. Family Practice 2004, 21 (6), 699-705

Mercer SW and Reynolds W J. Empathy and quality of care. BJGP 2002, 52 (Suppplement); S9-S12.

The CARE measure can be used free of charge. The Intellectual Property rights rest with the Scottish Executive. The measure may not be used on a commercial basis without the consent of the author and the Chief Scientist Office of the Scottish Executive Health Department, on behalf of the Scottish Ministers. If you would like more information, please contact;

Dr Stewart Mercer
General Practice and Primary Care, Division of Community-based Sciences,
University of Glasgow, 1Horselethill Road, Glasgow G12 9LX
Email; Stewmercer@blueyonder.co.uk

For further information, and to download the measure please visit;

http://www.gla.ac.uk/departments/generalpractice/caremeasure.htm
