# Supplementary material for: Effects of a 2-Week Remote Learning Program on Empathy and Clinical and Communication Skills in Premedical Students: Mixed Methods Evaluation Study
Source: JMIR Med Educ. 2021 Oct 27;7(4):e33090. doi: 10.2196/33090 (PMC8581748; doi:10.2196/33090)
Supplement: Multimedia Appendix 2 [file mededu_v7i4e33090_app2.pdf]

Pre-Survey Stanford Clinical Science, Technology and Medicine Summer Internship

---

Start of Block: Default Question Block

Q1 Please enter your unique ID number (provided by course staff):

____

End of Block: Default Question Block

Start of Block: Section 1: Empathy

Q11 What is empathy in the context of the patient-clinician setting?

____

____

____

____

____

Q12 Why is it important in the healthcare setting?

____

____

____

____

____

Q13 What are some ways to effectively demonstrate empathy?

Q14 Is it possible and effective to always be empathetic? Why or why not?

Q15 How might a clinician respond in the following scenarios?

	Response
When patients are rude	
When patients are vulnerable	
When patients do not listen because of previous knowledge they have about their condition	

End of Block: Section 1: Empathy

Start of Block: Section 2: Knowledge and Skills

Q5 Please rate the following statements about your specific knowledge and skills. Please mark one option for each statement and answer every statement.

	Strongly Disagree	Disagree	Neither agree nor disagree	Agree	Strongly agree
I can perform a basic surgical suture knot tie with effective closure for a skin wound.	○	○	○	○	○
I can demonstrate how to apply an epi pen to a person suffering from anaphylaxis effectively.	○	○	○	○	○
I can interpret a basic chest x-ray for signs of pneumothorax, hemothorax or major trauma.	○	○	○	○	○
When faced with a new challenge, I can analyze relevant information and creatively come up with potential solutions.	○	○	○	○	○
I can demonstrate effective communication skills to promote health.	○	○	○	○	○
I am capable of connecting and expanding on ideas when collaborating with peers.	○	○	○	○	○

I know how to listen to a friend who is sharing a problem.	○	○	○	○	○
I believe technology can play a crucial role in solving healthcare challenges.	○	○	○	○	○

End of Block: Section 2: Knowledge and Skills

Start of Block: Section 3: Background

Q6 What is the first initial of your first name?

____

Q7 What is the first initial of your last name?

____

Q8 What school year will you be entering in the fall?

____

Q9 What is your gender?

- ○ Male
- ○ Female
- ○ Other
- ○ Prefer not to say

Q10 What is your race?

- ○ American Indian or Alaska Native
- ○ Asian
- ○ Black
- ○ Native Hawaiian or Other Pacific Islander
- ○ White
- ○ More than one race
- ○ Other
- ○ Prefer not to say

End of Block: Section 3: Background
