# Supplementary material for: Effects of a 2-Week Remote Learning Program on Empathy and Clinical and Communication Skills in Premedical Students: Mixed Methods Evaluation Study
Source: JMIR Med Educ. 2021 Oct 27;7(4):e33090. doi: 10.2196/33090 (PMC8581748; doi:10.2196/33090)
Supplement: Multimedia Appendix 3 [file mededu_v7i4e33090_app3.pdf]

Post-Survey Stanford Clinical Science, Technology and Medicine Summer Internship

Start of Block: Default Question Block

Q1 Please enter your unique ID number (provided by course staff):

____

End of Block: Default Question Block

Start of Block: Section 1: Empathy

Q11 How has hearing stories from patients and other experts in the medical field influenced your view of medicine?

____

____

____

____

____

Q12 How has SASI helped shape your understanding of Everyone Included Principles?

____

____

____

____

____

Q21 Did you complete the Leadership and Engagement Track?

- ○ Yes
- ○ No

Skip To: End of Block If Did you complete the Leadership and Engagement Track? = No

Q22 How did the patient-physician role play activity shape your understanding of empathy?

____

End of Block: Section 1: Empathy

Start of Block: Section 2: Knowledge and Skills

Q5 Please rate the following statements about your specific knowledge and skills. Please mark one option for each statement and answer every statement.

	Strongly Disagree	Disagree	Neither agree nor disagree	Agree	Strongly agree
I can perform a basic surgical suture knot tie with effective closure for a skin wound.	○	○	○	○	○
I can demonstrate how to apply an epi pen to a person suffering from anaphylaxis effectively.	○	○	○	○	○
I can interpret a basic chest x-ray for signs of pneumothorax, hemothorax or major trauma.	○	○	○	○	○
When faced with a new challenge, I can analyze relevant information and creatively come up with potential solutions.	○	○	○	○	○
I can demonstrate effective communication skills to promote health.	○	○	○	○	○
I am capable of connecting and expanding on ideas when collaborating with peers.	○	○	○	○	○

I know how to listen to a friend who is sharing a problem.	○	○	○	○	○
I believe technology can play a crucial role in solving healthcare challenges.	○	○	○	○	○

Q13 What was the most important thing you learned as a SASI student?

____

____

____

____

____

Q14 What was your favorite activity or lecture, and why?

____

____

____

____

____

End of Block: Section 2: Knowledge and Skills

Start of Block: Section 3: Overall Feedback

Q15 Please respond to the following statement.

	Poor	Fair	Good	Very Good	Excellent
Please rate your overall SASI experience.	○	○	○	○	○

Q16 If you would like to, please elaborate on your answer above (optional).

____

____

____

____

____

Q17 How did SASI influence your interest in pursuing a career in healthcare?

____

____

____

____

____

Q18 What would you like to learn more about?

____

____

____

____

____

Q19 How can SASI be a better program? What was challenging for you?

____

____

____

____

____

End of Block: Section 3: Overall Feedback

Start of Block: Section 4: Background

Q6 What is the first initial of your first name?

____

Q7 What is the first initial of your last name?

____

X→

Q8 What grade will you be entering in the fall?

- 9th grade
- 10th grade
- 11th grade
- 12th grade
- College Freshman
- College Sophomore
- College Junior
- College Senior
- Other

Q9 What is your gender?

- Male
- Female
- Other
- Prefer not to say

Q10 What is your race?

- ○ American Indian or Alaska Native
- ○ Asian
- ○ Black
- ○ Native Hawaiian or Other Pacific Islander
- ○ White
- ○ More than one race
- ○ Other
- ○ Prefer not to say

End of Block: Section 4: Background
