# Supplementary material for: Effects of a 2-Week Remote Learning Program on Empathy and Clinical and Communication Skills in Premedical Students: Mixed Methods Evaluation Study
Source: JMIR Med Educ. 2021 Oct 27;7(4):e33090. doi: 10.2196/33090 (PMC8581748; doi:10.2196/33090)
Supplement: Multimedia Appendix 4 [file mededu_v7i4e33090_app4.pdf]

Stanford Clinical Science, Technology and Medicine Summer Internship Focus Group Questions

1. What is empathy in the context of the patient-clinician relationship?
2. Why is it important in healthcare?
3. What are some ways to effectively demonstrate empathy?
4. Is it possible and effective to always be empathetic? Why or why not?
    a. What/which factors might play a role in this?
5. How might a clinician respond given the following scenarios?
    a. When patients are rude
    b. When patients are vulnerable
    c. When patients do not listen because of previous knowledge they have about their condition
6. Is there anything else we have not covered in the previous questions that you would like to share with us?
